# Exploring photosynthesis evolution by comparative analysis of metabolic networks between chloroplasts and photosynthetic bacteria

**DOI:** 10.1186/1471-2164-7-100

**Published:** 2006-04-30

**Authors:** Zhuo Wang, Xin-Guang Zhu, Yazhu Chen, Yuanyuan Li, Jing Hou, Yixue Li, Lei Liu

**Affiliations:** 1 Biomedical Instrument Institute, Shanghai Jiao Tong University, 1954 Huashan Rd, Shanghai, 200030, China; 2 Department of Plant Biology, University of Illinois at Urbana-Champaign, 1201 W. Gregory Dr., Urbana, Illinois 61801, USA; 3 The W. M. Keck Center for Comparative and Functional Genomics, University of Illinois at Urbana-Champaign, 1201 W. Gregory Dr., Urbana, Illinois 61801, USA; 4 Shanghai Center for Bioinformation Technology, 100 Qinzhou Rd, 12^th ^Floor, Shanghai, 200235, China; 5 Department of Computer Engineering and Science, Shanghai University, 149 Yanchang Rd, Shanghai, 200072, China

## Abstract

**Background:**

Chloroplasts descended from cyanobacteria and have a drastically reduced genome following an endosymbiotic event. Many genes of the ancestral cyanobacterial genome have been transferred to the plant nuclear genome by horizontal gene transfer. However, a selective set of metabolism pathways is maintained in chloroplasts using both chloroplast genome encoded and nuclear genome encoded enzymes. As an organelle specialized for carrying out photosynthesis, does the chloroplast metabolic network have properties adapted for higher efficiency of photosynthesis? We compared metabolic network properties of chloroplasts and prokaryotic photosynthetic organisms, mostly cyanobacteria, based on metabolic maps derived from genome data to identify features of chloroplast network properties that are different from cyanobacteria and to analyze possible functional significance of those features.

**Results:**

The properties of the entire metabolic network and the sub-network that consists of reactions directly connected to the Calvin Cycle have been analyzed using hypergraph representation. Results showed that the whole metabolic networks in chloroplast and cyanobacteria both possess small-world network properties. Although the number of compounds and reactions in chloroplasts is less than that in cyanobacteria, the chloroplast's metabolic network has longer average path length, a larger diameter, and is Calvin Cycle -centered, indicating an overall less-dense network structure with specific and local high density areas in chloroplasts. Moreover, chloroplast metabolic network exhibits a better modular organization than cyanobacterial ones. Enzymes involved in the same metabolic processes tend to cluster into the same module in chloroplasts.

**Conclusion:**

In summary, the differences in metabolic network properties may reflect the evolutionary changes during endosymbiosis that led to the improvement of the photosynthesis efficiency in higher plants. Our findings are consistent with the notion that since the light energy absorption, transfer and conversion is highly efficient even in photosynthetic bacteria, the further improvements in photosynthetic efficiency in higher plants may rely on changes in metabolic network properties.

## Background

Photosynthesis is one of the most important and fundamental metabolic processes in the biosphere. The appearance of photosynthesis in prokaryotic organisms early in the earth's history fundamentally changed the composition of the atmosphere and subsequently determined the evolution of organisms. According to the theory of endosymbiosis, chloroplasts descended from cyanobacteria [1,2]. During endosymbiosis, the ancestral cyanobacterial genome was drastically reduced, and many genes were transferred to the nuclear genome [1,3]. As a result, the majority of the enzymes in chloroplast metabolic networks are nucleus-encoded, translated in cytosol, and then imported into chloroplasts [4]. Such massive transportation of proteins requires a large amount of energy and sophisticated regulation from plant cells. Since the metabolic networks in chloroplasts are mostly constructed with proteins encoded in nuclear genome, do the networks exhibit some unique properties and characteristics that deviate from the ancestors' metabolic networks? To answer this question, we conducted a comparative study of the metabolic networks between chloroplasts and several photosynthetic bacteria.

Studies on the evolution of photosynthesis have mostly focused on individual proteins or protein complexes related to photosynthesis [1,5-7]. With the recent advancements in genomics and the development of metabolic pathway databases, we are now able to reconstruct metabolic networks from complete and annotated genomes and conduct system-level comparisons of the metabolic networks. Recently, there have been several such studies comparing system-wide network properties among many organisms [8,9]. In this study, we examined the similarity and differences of network properties between chloroplasts and the photosynthetic bacteria including connectivity, clustering coefficient, path length, network diameter [8,9], and modularity [10-13]. Comparisons of modular structures of the metabolic network provide insights about the modification of major metabolisms of chloroplasts, such as addition or loss of certain metabolisms and the changes in the organization of metabolism due to endosymbiosis.

## Results

### Chloroplast metabolic network exhibits different characteristics compared to photosynthetic bacteria

The basic statistics of reconstructed metabolic networks in chloroplasts and photosynthetic bacteria are shown in Table 1. The numbers of enzymes in all metabolic networks were similar. However, there were more cases of one enzyme catalyzing two or more reactions in the photosynthetic bacteria. For example, aminomethyltransferase (EC 2.1.2.10) catalyzes three reactions in *synechorocus sp*. WH8102 (syw):

Glycine + Tetrahydrofolate + NAD^+ ^<=> 5,10-Methylenetetrahydrofolate + NH_3 _+ CO_2 _+ NADH + H^+^

5-Formyltetrahydrofolate <=> 5,10-Methenyltetrahydrofolate + H_2_O

Tetrahydrofolate + S-Aminomethyldihydrolipoylprotein <=> 5,10-Methylenetetrahydrofolate + NH_3 _+ Dihydrolipoylprotein

In contrast, only the last reaction exists in the chloroplast network. When we compared enzymes in chloroplasts and photosynthetic bacteria, we found some differences among them. For example, there are 376 and 371 enzymes respectively in chloroplast and *Synechococcus sp*. WH8102 (syw) metabolic network, among which 210 enzymes are shared by them. The complete list of enzymes of chloroplasts, photosynthetic bacteria, *E.coli*, *Arabidopsis thaliana and Cyanidioschyzon merolae *are all listed in Additional file 1.

Even though the numbers of compounds and reactions in chloroplast network are fewer than those in photosynthetic bacteria, the average connectivity of compound nodes is very similar among them (Table 1). In addition, the distribution of compound connectivity in chloroplasts and cyanobacteria followed the Power law (see Additional file 2). The average clustering coefficients, the average path lengths and the diameters of both enzyme and compound nodes (Table 1) confirmed that the metabolic networks under study are scale-free and small-world networks using hypergraph model. It is evident from Table 1 that the topological properties are very similar among all photosynthetic bacteria, while chloroplasts exhibit some differences. Although the chloroplast network has fewer compound nodes and hyper-edges in its hypergraph representation, the average path lengths and diameters of both enzyme and compound nodes are longer than those in photosynthetic bacteria. The average clustering coefficient of both enzyme and compound nodes are lower in chloroplasts, suggesting an overall loose network structure in chloroplast. We also conducted an in-depth comparison of the densities of enzyme networks in chloroplasts and cyanobacteria by analyzing the cores using Pajek [14]. The k-core of a network is defined as a subnetwork of a given network where each vertex has at least k neighbors in the same core. For chloroplasts and *Synechococcus sp*. WH8102 (syw), the largest core includes 32 and 37 enzymes respectively, among which 24 enzymes are shared by the two cores.

### The network is highly clustered around Calvin Cycle in chloroplasts

**Figure 1:**
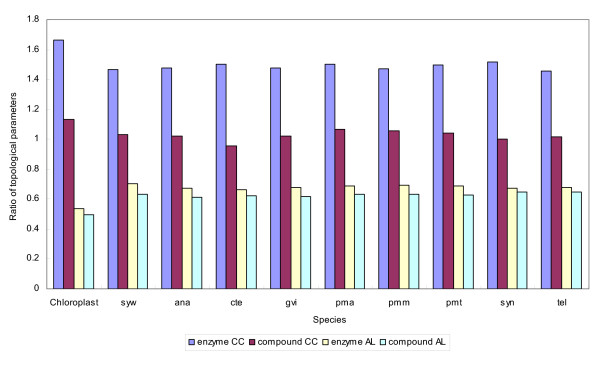
Ratio of topological properties in SubNetwork to whole network for chloroplasts and photosynthetic bacteria. CC: clustering coefficient; AL: average path length.

For the SubNetwork, which includes reactions directly connected with the Calvin Cycle, the average clustering coefficient is higher and the average path length is shorter than the whole network, indicating tighter linkage between reactions in the SubNetwork, in both chloroplast and photosynthetic bacteria (see Additional file 3). Although the overall chloroplast network shows a lower average clustering coefficient and longer average path length compared to photosynthetic bacteria, the ratio of average clustering coefficient between the SubNetwork and the whole network is higher in chloroplasts than that in photosynthetic bacteria. The ratio of average path length between the SubNetwork and whole network is lower in chloroplasts than that in photosynthetic bacteria (Figure 1), suggesting that the chloroplast network is highly clustered around the Calvin Cycle.

Furthermore, we made an interesting observation when we ranked the connectivity of different compounds in the network. We extracted the top ten connected (hub) compounds in the whole network and then checked their ranks in the SubNetwork. It is interesting to notice that glutamate, which is a crucial compound for nitrogen assimilation, is highly connected (hub) in the whole networks of both chloroplast and cyanobacteria. However, glutamate does not exist in the chloroplast SubNetwork but still exists in all cyanobacteria SubNetworks. The difference lies in the reaction L-Glutamate <=> 4-Aminobutanoate + CO_2 _catalyzed by L-Glutamate 1-carboxy-lyase (EC 4.1.1.19), which is missing in chloroplast. This observation suggests that the nitrogen assimilation is not directly linked to carbon fixation in chloroplasts, but is linked in cyanobacteria.

### Simulation of the possible impact of an incomplete dataset on the topological properties of metabolic network

Most data collected in this study were originated from genome annotations, which may be incomplete. In order to assess the effect of such incomplete data, we designed an experiment using the well-studied and most complete *E. coli *metabolic network. First, the topological properties of the entire network were calculated using the hypergraph model. Then, fractions of enzymes and reactions were randomly removed from the network and the network properties were again calculated. The results after random removal of nodes were used to simulate the impact of incomplete metabolic information on the full network. Table 2 demonstrates that the topological properties of the metabolic network remain nearly unaffected when 35% of the enzymes were randomly removed. Even after removal of 50% the topological parameters change by less than 5% from those of the complete network. The diameters increase by 8.33% over the original network, which represents the most significantly changed parameter, but this value is far lower than the differences of network parameters between chloroplasts and photosynthetic bacteria, indicating that the topological differences of the two networks are unlikely to be caused by an incomplete dataset. These results strongly validate the significance of our comparisons between chloroplasts and photosynthetic bacteria and support the conclusion that chloroplasts have an overall loose but strongly Calvin Cycle-centered network structure.

### The chloroplast network shows a better modular structure than photosynthetic bacteria

A natural step after the study of overall properties of a complex network is to investigate the substructures within the network and possible functions of the substructures. One of the methods to decompose a complex network structure is to find modules within the network based on the connectivity among the nodes. In this study, we view modules as sub-networks where the nodes are highly connected within a module, but much less connected between modules.

Many approaches have been used to detect modules in metabolic network including elementary modes, extreme pathways, flux analysis [15-17], and graph clustering techniques such as Markov Clustering [MCL, 18], Iterative Conductance Cutting [ICC, 19], and Geometric Minimal Spanning Tree Clustering [GMC, 20]. After comparison, we adopted the method from Guimerà and Amaral [21,22] to identify modules in metabolic networks in chloroplasts and photosynthetic bacteria (see detailed description in the "Methods" section). This method is called the SA module-detection algorithm in the remainder of the text.

**Figure 2:**
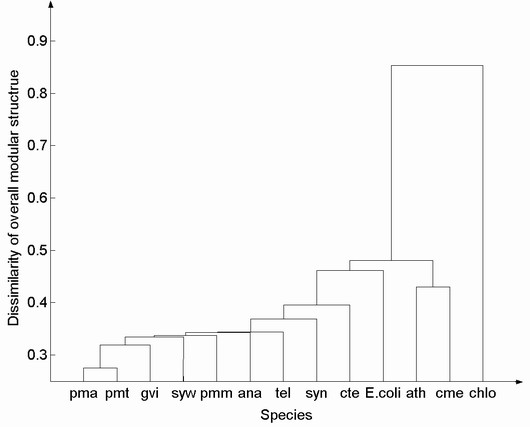
Similarity of overall modular structures among chloroplasts, photosynthetic bacteria, *E.coli*, *Arabidopsis thaliana *and *Cyanidioschyzon merolae*.

Modular structures differ among different organisms. The similarity of overall modular structure among chloroplasts, photosynthetic bacteria, *E.coli*, *Arabidopsis thaliana and Cyanidioschyzon merolae *has been calculated and is shown as a dendrogram in Figure 2 (see "Methods" section for detailed description of the similarity measurements of modules). Remarkably, all cyanobacteria exhibit very similar modular organization and are different from chloroplasts. Arabidopsis thaliana and Cyanidioschyzon merolae are clustered together with high similar modular structure. This result is consistent with the topological results (Table 1) that chloroplast metabolic network shows different characteristics.

**Figure 3:**
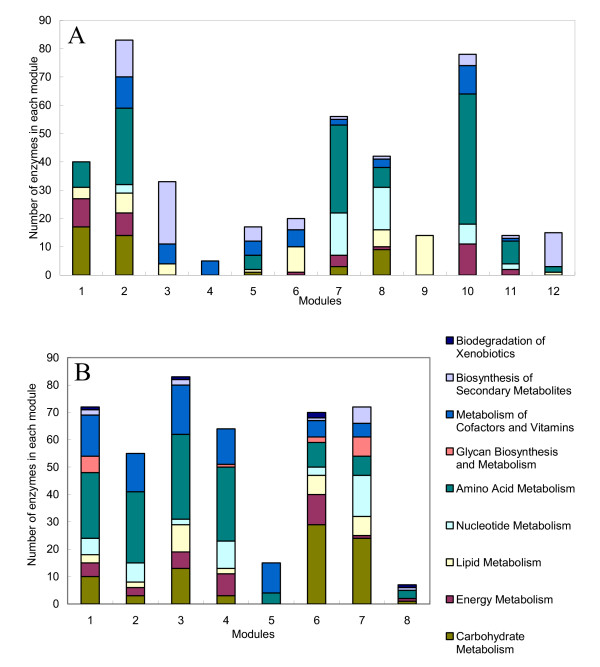
Comparison of functional modules in chloroplasts and cyanobacteria. (A) Chloroplast enzyme modules; (B) *Synechococcus sp*. WH8102 (syw) enzyme modules map according to KEGG classification.

Matching modules to particular metabolisms reveals the possible biological significance of modularity [21,22]. The function of each enzyme module in chloroplast and photosynthetic bacteria was classified using the classification scheme proposed in KEGG which includes nine major pathways: carbohydrate metabolism, energy metabolism, lipid metabolism, nucleotide metabolism, amino-acid metabolism, glycan biosynthesis and metabolism, metabolism of cofactors and vitamins, biosynthesis of secondary metabolites, and biodegradation of xenobiotics. Based on Guimerà and Amaral [21,22], we mapped the modules to KEGG functional classifications; if more than 50% of the enzymes in a module belong to one major pathway, then the module is considered pathway specific. The match between modules and KEGG classifications for chloroplasts and *Synechococcus sp*. WH8102 (syw) are shown in Figure 3. Other cyanobacteria showed similar functional categories mapping to their corresponding modules. Interestingly, glycan biosynthesis and metabolism, and biodegradation of xenobiotics are absent in chloroplasts but present in cyanobacteria (Figure 3A,B). In addition, some metabolic processes related to gibberellins, abscisic acid, brassinolide, cytokinin, indole-3-acetic acid, ethylene, polyamine and jasmonic acid are specific to chloroplasts, which are mostly included in module 3. Most of these molecules are related to hormone synthesis or metabolism [23-25].

Several modules were organized around amino-acid metabolic functions in both chloroplasts and *Synechococcus sp*. WH8102 networks, which are module 2, 7, 10, 11 in chloroplast and module 1, 2, 3, 4 in *Synechococcus sp*. WH8102, respectively. In chloroplasts, module 4 exclusively consists of enzymes in cofactor and vitamin metabolism, and all enzymes in module 9 belong to lipid metabolism (Figure 3A). However no module in *Synechococcus sp*. WH8102 completely corresponds to any one specific pathway (Figure 3B). Nearly 90% of the enzymes in module 3 in the chloroplast network are related to biosynthesis of secondary metabolites. Also 80% enzymes in module 12 relate to hormone metabolism in chloroplasts (Figure 3A). In contrast, only module 5 and module 8 in the cyanobacteria contain more than 50% enzymes belonging to cofactor and vitamin metabolism and to amino acid metabolism respectively (Figure 3B).

**Figure 4:**
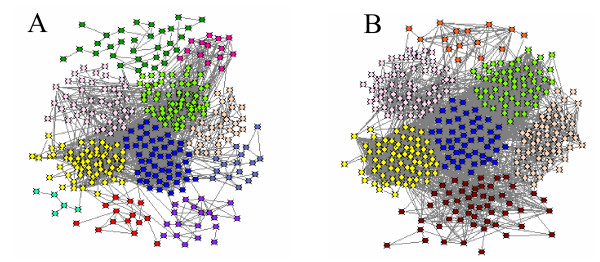
Conserved and different modules in metabolic network between chloroplasts and *Synechococcus sp*. WH8102 (syw). The modular structures of enzyme-centric networks for chloroplasts and syw are shown in (A) and (B) respectively. Each module is represented by a specific color. The five pairs of modules with same color are conserved modules between chloroplast and syw, among which the yellow, green and blue modules correspond to amino-acid metabolism, the light-orange and pink modules belong to carbohydrate metabolism and nucleotide metabolism respectively. The picture was drawn using the Pajek program.

By comparing the similarity between any two modules in chloroplasts and each photosynthetic bacterium, we found for each bacterium 5 to 7 modules similar to corresponding modules in chloroplasts. Moreover five pairs of these modules are very conserved among chloroplasts and photosynthetic bacteria: three pairs correspond to amino-acid metabolism, two pairs belong to carbohydrate metabolism and nucleotide metabolism respectively, all of which are related to the core metabolism. It is evident that the core metabolic processes are conserved in evolution. As an example, the comparison of modules between chloroplast and *Synechococcus sp*. WH8102 was visualized in Figure 4. The five modules with the same color are composed of similar enzymes, mapped to the same functional pathways. These five conserved modules include 69.68% and 80.32% of all enzymes in chloroplasts and *Synechococcus sp*. WH8102, respectively. Of the common 210 enzymes between chloroplasts and *Synechococcus sp*. WH8102, approximately 60% of them exist in the conservative modules. The other modules in chloroplasts mainly correspond to metabolism of cofactors and vitamins, and biosynthesis of secondary metabolites. This result indicates that the core metabolisms of chloroplasts are similar to cynobacteria, including carbohydrate metabolisms, amino acid metabolisms and nucleotide metabolism. The difference lies on the specialized pathways.

## Discussion

This study showed that the chloroplast metabolic network is less dense in comparison to photosynthetic bacteria as indicated by longer path length, larger diameter and fewer reactions. It has been suggested by Ma and Zeng [6] that the three domains of organisms exhibit quantitative differences in the metabolic network properties, i.e. eukaryotes and archaea seem to have a longer path length and a larger network diameter than bacteria. Our results suggest that global properties of chloroplast metabolic network are closer to eukaryotes than to bacteria, which may be a result of re-construction of metabolic networks by most of nucleus-coded proteins.

When comparing the SubNetwork properties, the chloroplast network is highly centered around the Calvin Cycle, indicating that the chloroplast network appears to be simplified on one hand but highly specialized on the other. This notion is further echoed by the subsequent investigation on modular structures (see below). The results could also support a view that the highly developed apparatus of light energy harvesting and its conversion to chemical energy has been optimized in cyanobacteria and that further metabolic advantages could be gained by improving the carbon fixation reactions in higher plants. Evolution of the different enzymes involved in photosynthesis has been studied extensively [26]. Our study suggests that overall network properties could be an addition to the phylogenetic analysis of individual enzymes, and might provide more information about the evolutionary history of chloroplasts.

In addition to being overall loose and Calvin Cycle-centered, chloroplast metabolic network shows a better modular structure than that of photosynthetic bacteria by SA module-detection algorithm. Our results showed that seven of the chloroplast modules are very pathway-specific in that more than 50% of the enzymes in the module belong to one pathway, such as amino acid synthesis, or carbohydrate metabolism (Figure 3A). In contrast, of the eight modules detected in *Synechococcus sp*. WH8102, only two modules show such pathway-specificity (Figure 3B). Moreover, two modules in chloroplasts are composed of enzymes of two pathways exclusively, lipid metabolism and the metabolism of cofactors and vitamins. Clearly, chloroplast metabolic network exhibits very different modular structure compared to cyanobacteria. Modules detected in this study represent the grouping of reactions based on their connections, which reflect in some degree the coordination of the whole metabolism. In chloroplasts, the overall complexity of the metabolic network seems reduced with fewer reactions and absence of some pathways, but the network becomes more organized with a highly modular structure.

All of the nine KEGG pathways exist in photosynthetic bacteria while two of them, glycan biosynthesis and biodegradation of xenobiotics, are absent in chloroplast. These two pathways are present in the cytosol of plant cells. Glycan biosynthesis, which underlines the synthesis of cellulose and glycol-protein on cell walls, is energetically favored to reside in cytosol instead of chloroplasts. If glycan synthesis resided in chloroplasts, the transfer of glycan from chloroplast to cell wall would need substantial energy input. Xenobiotic degradation is mostly carried out in peroxisomes in plant cells [27]. As the site of photosynthesis and O_2 _release, chloroplast stroma generate superoxide radicals [28], which could be a good place for xenobiotic degradation. However, these superoxides in chloroplast stroma would react with xenobiotics or xenobiotic degradation intermediates and form toxic radicals, which require a better control and subsequently reduce the efficiency of photosynthesis. Obviously, the compartmentalization of eukaryotic cells causes the specialization of functions and increase of efficiency in organelles. We also notice that metabolic processes related to hormones exist in chloroplasts, but not in any photosynthetic bacteria. It is quite intuitive that as multi-cellular organisms, plants need to communicate between cells. Hormones are the means of such communication. Those reactions related to hormones are probably a result of later addition from higher plants.

Despite the differences, some of the pathways are conserved between chloroplasts and photosynthetic bacteria. We noticed that five modules are common among all species in the study, which form a core of metabolism including carbohydrate metabolism, amino acid metabolism, and nucleotides metabolism. But the organization of these modules is different between chloroplasts and photosynthetic bacteria. The modules in chloroplasts show higher functional specificity than their counterparts in photosynthetic bacteria. The modules in photosynthetic bacteria appear to have a mixture of functions. For example, the Calvin Cycle is completely embedded in one module in chloroplasts, but split into two modules in *Synechococcus sp*. WH8102.

Recent studies have shown that cellular evolution might have been mainly driven by horizontal gene transfer (HGT) [29,30]. Since the metabolic network of chloroplasts exhibits a more highly modular organization, its evolution may be a result of multiple HGTs. In fact, multiple horizontal gene transfer events have been implied through the phylogenetic analysis of the key proteins involving photosynthetic light reactions [26]. Martin et al. found 1700 cyanobacteria genes in *Arabidopsis *nucleus including 166 genes with EC numbers, among which 92 enzymes are targeted to chloroplasts [3]. We mapped these 92 enzymes to modules in the chloroplast network and found 88% of the enzymes exist in the conserved modules corresponding to the core metabolism. The highly modular structure of chloroplast metabolism is possibly a prerequisite for a higher photosynthetic efficiency because a high modular structure can response to environmental or internal changes in a more coordinated and robust way. From another perspective, the light energy harvesting, transfer, and conversion to chemical energy in the form of ATP and NADPH has reached a high efficiency even in cyanobacteria [31,32]. As a result, changes in metabolic stoichiometry, in addition to changes in enzyme kinetics of certain key enzymes such as Rubisco [33] might represent the available options for higher photosynthetic efficiency. In this aspect, this is consistent with the results that chloroplast metabolism is centered on the Calvin Cycle.

## Conclusion

In summary, by comparing the topological properties and features of metabolic networks between chloroplasts and photosynthetic bacteria, we showed that the chloroplast metabolic networks are reduced and simplified on one hand, but highly specialized and modular on the other. While overall density of the metabolic network in chloroplasts is reduced comparing to photosynthetic bacteria, the density of sub-networks directly linked to Calvin Cycle is increased. The chloroplast metabolic network also exhibits a highly modular structure compared to the metabolic network of photosynthetic bacteria. These special features of chloroplast metabolic network may reflect changes in the reconstruction of the network during endosymbiosis and the results of horizontal gene transfer. Functional mapping of the modules revealed that chloroplast metabolic network exhibited high functional specificity to the modules, indicating a better coordination of the overall metabolism and specialization of functions. Our findings are consistent with the notion that since the light energy absorption, transfer and conversion is highly efficient even in photosynthetic bacteria, the further improvements in photosynthetic efficiency in higher plants may rely on changes in metabolic network properties.

## Methods

### Dataset preparation

**Figure 5:**
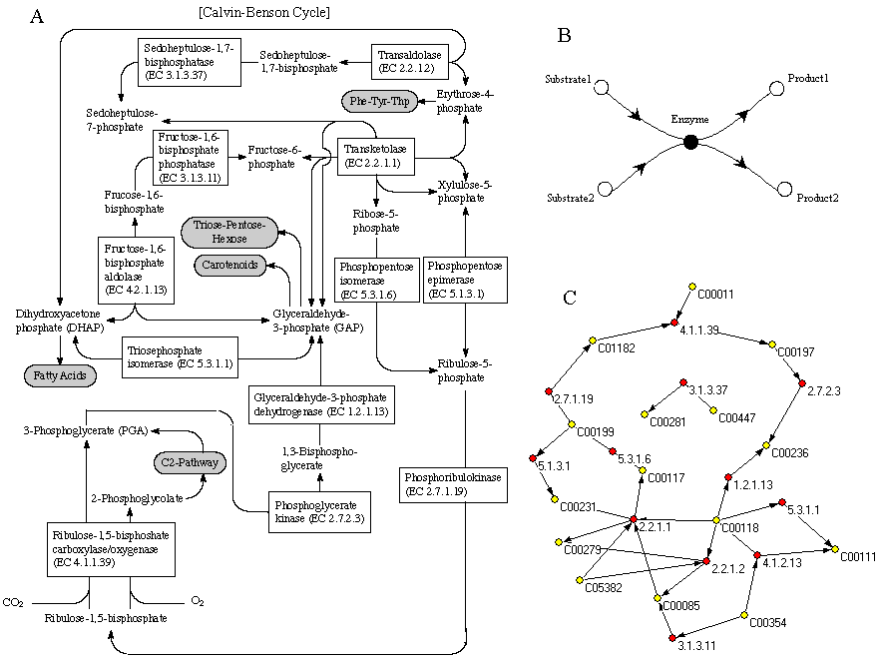
The Calvin Cycle pathway and its hypergraph representation.(A) The metabolic scheme of the Calvin Cycle, derived from the Database of Chloroplast/Photosynthesis Related Genes. (B) An example of hypergraph representation of biochemical reactions. (C) Graph visualization of the Calvin Cycle pathway in (A), where the red nodes and yellow nodes represent enzymes and compounds respectively. ATP, ADP, H2O, H^+^, NAD^+^, NADP^+^, NADH, NADPH, Orthophosphate and Pyrophosphate have been omitted.

The metabolic pathway data for chloroplasts were extracted from the Database of Chloroplast/Photosynthesis Related Genes collected by the Nagoya Plant Genome Group [34], which is a general dataset including all chloroplast enzymes in several plants, such as *Arabidopsis thaliana*, *Oryza sativa *and tobacco. For photosynthetic bacteria, we extracted the metabolic networks of nine species from KEGG: *Anabaena sp*. PCC7120 (ana), *Chlorobium tepidum (cte)*, *Gloeobacter violaceus (gvi)*, *Prochlorococcus marinus *SS120 (pma), *Prochlorococcus marinus *MED4 (pmm), *Prochlorococcus marinus *MIT9313 (pmt), *Synechocystis sp.* PCC6803 (syn), *Synechococcus sp*. WH8102 (syw), *Thermosynechococcus elongates (tel)*. We also collected the metabolic pathways of *E.coli*, *Arabidopsis thaliana* and *Cyanidioschyzon merolae (red algae) *from KEGG. We coded enzymes and compounds by their corresponding EC number and compound ID number in the KEGG database, respectively. The direction of reactions was obtained based on the rules provided by Ma and Zeng [6]. A sub-network was constructed by including all reactions sharing metabolites with the Calvin Cycle. All enzymes and reactions in the Calvin Cycle are shown in Figure 5A.

### Network reconstruction and topological properties of networks

Most metabolic reactions have more than one substrate and/or more than one product, and therefore violate the condition of a one-to-one relationship between vertices and edges of a simple graph. Here we used a hypergraph model [35,36] to represent metabolic networks, where a hyper-edge represents a reaction and nodes represent different components involved in the reaction (i.e. enzymes and compounds). The hyper-edge relates a set of substrates to a set of products via enzymes. Figure 5B gives an example of a hypergraph, which offers an unambiguous representation of the enzymes and compounds in biochemical networks. The topological properties of both enzymes and compounds can be represented and analyzed simultaneously. The following topological properties were calculated:

#### Connectivity (degree)

The connectivity of an enzyme node A is defined as the number of enzymes sharing compounds with the reaction catalyzed by A. For example, in Figure 5C, Fructose-1,6-bisphosphate phosphatase (3.1.3.11) catalyzes one reaction including two compounds C00354 and C00085. There are three enzymes catalyzing reactions sharing these two compounds, which are: fructose-1,6-bisphosphate aldolase (4.1.2.13), Transaldolase (2.2.1.2) and Transketolase (2.2.1.1). Therefore, the connectivity of Fructose-1,6-bisphosphate phosphatase (3.1.3.11) is three. The connectivity of a compound node is the number of hyper-edges containing the given compound. Average enzyme connectivity and compound connectivity are computed by averaging these two properties over all enzyme or compound nodes, respectively.

#### Path length

Path length is the number of hyper-edges in the shortest path connecting two enzyme nodes or compound nodes. For example, in Figure 5C, the path length from C00354 to C00279 is two. The average path length (AL) of the entire hypergraph is the path length between each of two nodes, averaged over all pairs of nodes.

#### Diameter

The diameter of a hypergraph is the maximum path length between any pair of nodes.

#### Clustering coefficient

This parameter measures the "cliquishness" of the neighborhood of a given node. Assuming *k *nodes are connected to a given node *v *and there are *m *hyper-edges between these *k *nodes (not including hyper-edges connecting them to *v*), the clustering coefficient of node *v *is: *C(v) = 2 m/[k(k-1)]*. For example, Fructose-1,6-bisphosphate phosphatase (3.1.3.11) has three enzymes connected to it, and every two of these three are connected, so *m *is 3 and *C(v) *is 1 for this enzyme. The clustering coefficient (CC) of all enzyme or compound nodes in the hypergraph is defined as the average of *C(v) *over all enzyme or compound nodes.

Some small molecules, such as adenosine triphosphate (ATP), adenosine diphosphate (ADP), nicotinamide adenine dinucleotide (NAD) and H_2_O, are normally used as carriers for transferring electrons or energy and participate in many reactions, while typically not participating in product formation. The connections through these compounds should be treated differently when calculating the path length from one metabolite to another. The following small molecules were disregarded in the calculations as well as their connections when no product was formed: ATP, ADP, H_2_O, H^+^, NAD^+^, NADP^+^, NADH, NADPH, Orthophosphate, and Pyrophosphate,. It should be noted that the omission is not determined by the compound, but by the reaction. For example, H_2_O is a small metabolite in many reactions, but in the following reaction:Putrescine + Oxygen + H_2_O <=> 4-Aminobutanal + NH_3 _+ H_2_O_2_, H_2_O cannot be omitted because it participates in producing H_2_O_2_.

The Calvin Cycle is a key pathway in photosynthesis. We have defined the SubNetwork as a sub-network directly linked to the Calvin Cycle using the reactions that share all the compounds in the Calvin Cycle, with the exception of the small molecules listed before. We calculated the network properties of the SubNetwork and the ratios of each property between the SubNetwork and the total network.

### Module discovery of enzyme-centric graphs

Module discovery methods based on metabolic flux are either intractable at the genome scale or have more overlap between modules [15-17]. The graph clustering techniques are regarded as appropriate for network modules detection; experimental study confirms MCL performs better than ICC and GMC in many cases [37]. In general, the MCL algorithm performs well for graph clustering except for graphs which are very homogeneous (such as weakly connected grids) and for graphs in which the natural cluster diameter (i.e. the diameter of a subgraph induced by a natural cluster) is large [38]. It has been successfully adapted to protein family classification, which has rather complete and definite data. However, MCL often gives a trivial clustering and is sensitive to signal noise, which may generate biologically insignificant modules. Guimerà and Amaral [21,22] identify modules in metabolic networks by maximizing the network's modularity using simulated annealing. By relating the metabolites in any given module to KEGG's nine major pathways, they validated that more than one-third of the metabolites in any module belong to a single pathway, which can provide a functional cartographic representation of the complex network.

We compared the modularity of metabolic networks by MCL and SA, and found MCL generated more small-size modules compared to SA, which were difficult to map to higher level functional categories. MCL decomposed the chloroplast enzyme network into 48 modules and the photosynthetic bacteria network into 30–40 modules. The size of the modules exhibits a power-law distribution, where one or two large modules include many enzymes from several unrelated biological pathways and many modules only consist of no more than four enzymes. SA, in contrast, gives a moderate number of modules. The SA algorithm detected 12 modules in the chloroplast enzyme network and 8 to 9 modules for the photosynthetic bacterial species. Each module consists of enzymes involved in one or several particular metabolic functions. A detailed list of enzymes in each module by both MCL and SA in all species can be seen in Additional file 4, 5, 6, 7, 8, 9, 10, 11, 13. This comparison indicates that SA might be more appropriate for the clustering analysis in this study. We selected modules detected by SA algorithm for similarity analysis and functional classification.

Deviating from Guimerà and Amaral [21,22], we used an enzyme-centric graph representation of the metabolic network where vertices were used to represent enzymes and edges were used to represent compounds. There will be a directed edge from enzyme *E*_1 _to enzyme *E*_2_, if *E*_1 _catalyzes a reaction generating a product *A *which is used as substrate of *E*_2_. Reversible reactions are considered as two separate reactions. Modularization of such enzyme-centric graph categorizes enzymes into different functional groups.

### Similarity measure of modular structures

To compare the modular structures among the networks from different species, we define a similarity measure based on Hamming distance [39]. For two modules *a *and *b *in two species, the number of enzymes in each module is *N*_*a *_and *N*_*b*_. First, we compute the similarity between any two enzyme members between module *a *and *b*. Any EC number is treated as a vector with 4 parts, which are given different weight 0.1, 0.2, 0.3, 0.4 according to EC hierarchy. For two EC numbers, one vector *P *emerges to describe their similarity. If they are same at the *k*th level, then *P*_*k *_is 1, otherwise *P*_*k *_is 0. Thus the similarity between any two enzymes *i *and *j *is defined as:



Note that the comparison of two EC numbers should be from high level to low level, if different at the *k*th level, then all *P*_*t *_(*t*>=*k*) will be 0 regardless of whether they are the same at lower levels.

After collecting all similarities between any two enzymes, the most similar enzyme in module *b *for each enzyme *i *in module *a *is identified. This maximal similarity is represented as *Sbest*_*i*_. Then the global similarity between module *a *and module *b *should be defined as:



Therefore, for any module in one species, its most similar module in another species can be identified. If two modules of two species are both most suited each other, they are regarded as conserved modules between these two species.

In order to investigate the overall modular structure among different species, we compared the modular similarity between two species based on the similarity between modules. Each module in each species is regarded as a sample, and the total of these samples as a large group. Thus the similarity between two species can be measured by the similarity between these two groups, which is defined according to the Hausdorff metric [40]. *G*_1 _and *G*_2 _are two groups representing two species, *S*_*species*_*(G*_1_*,G*_2_*) *is the similarity between these two species, *a *and *b *are samples (modules above) belonging to *G*_1 _and *G*_2_, respectively. The similarity *S(a, G*_2_*) *between sample *a *belonging to group *G*_1 _and group *G*_2 _is defined as:



Then, the similarity between *G*_1 _and *G*_2 _is given by:



It is important to note that this similarity is in general not symmetrical. Accordingly we introduce the similarity between *G*_2 _and *G*_1_:



It is then convenient to introduce the similarity between two species as:

S_*species*_(G_1_,G_2_) = min{S(G_1_,G_2_),S'(G_2_,G_1_)}

The Hausdorff metric provides a more accurate measurement of the structure similarity between two species, since the lower value of the forward and backward similarity is selected, which leads to a significantly underestimated assessment.

## Authors' contributions

ZW conducted the analysis of network properties and module discovery and implemented programs for the analysis. XGZ designed the experiments and analysis. YZC managed the project. YYL provided biological analysis. JH implemented software programs for the module comparison. YXL managed the project. LL designed and managed the project. All authors read and approved the final manuscript.

## Supplementary Material

Additional File 1 The complete list of enzymes of chloroplasts, photosynthetic bacteria, *E.coli*, *Arabidopsis thaliana and Cyanidioschyzon merolae*. Click here for file

Additional File 2 The distribution of compound connectivity in chloroplasts and photosynthetic bacteria. Click here for file

Additional File 3 The constitution and topological properties of sub-network directly connected with the Calvin Cycle in chloroplasts and photosynthetic bacteria. Click here for file

Additional File 4 The detailed list of enzymes in each module by both MCL and SA in metabolic network of chloroplasts, ana, cte, gvi, pma, pmm, pmt, syn, syw, tel. Click here for file

Additional File 5 The detailed list of enzymes in each module by both MCL and SA in metabolic network of chloroplasts, ana, cte, gvi, pma, pmm, pmt, syn, syw, tel. Click here for file

Additional File 6 The detailed list of enzymes in each module by both MCL and SA in metabolic network of chloroplasts, ana, cte, gvi, pma, pmm, pmt, syn, syw, tel. Click here for file

Additional File 7 The detailed list of enzymes in each module by both MCL and SA in metabolic network of chloroplasts, ana, cte, gvi, pma, pmm, pmt, syn, syw, tel. Click here for file

Additional File 8 The detailed list of enzymes in each module by both MCL and SA in metabolic network of chloroplasts, ana, cte, gvi, pma, pmm, pmt, syn, syw, tel. Click here for file

Additional File 9 The detailed list of enzymes in each module by both MCL and SA in metabolic network of chloroplasts, ana, cte, gvi, pma, pmm, pmt, syn, syw, tel. Click here for file

Additional File 10 The detailed list of enzymes in each module by both MCL and SA in metabolic network of chloroplasts, ana, cte, gvi, pma, pmm, pmt, syn, syw, tel. Click here for file

Additional File 11 The detailed list of enzymes in each module by both MCL and SA in metabolic network of chloroplasts, ana, cte, gvi, pma, pmm, pmt, syn, syw, tel. Click here for file

Additional File 12 The detailed list of enzymes in each module by both MCL and SA in metabolic network of chloroplasts, ana, cte, gvi, pma, pmm, pmt, syn, syw, tel. Click here for file

Additional File 13 The detailed list of enzymes in each module by both MCL and SA in metabolic network of chloroplasts, ana, cte, gvi, pma, pmm, pmt, syn, syw, tel. Click here for file

## Figures and Tables

**Table 1:** Structure and topological properties of whole network in chloroplasts and several photosynthetic bacteria.

Species	Enzyme number	Compound number	Reaction number	Average compound connectivity	Enzyme CC	Compound CC	Enzyme AL	Compound AL	Enzyme Diameter	Compound Diameter
Chloroplast	376	586	560	2.6185	0.534371	0.431872	5.07847	4.83902	19	19
syw	371	860	694	2.5615	0.59365	0.503954	4.07523	3.972854	11	12
ana	401	881	728	2.5182	0.590467	0.513945	4.15901	3.95608	11	12
cte	323	724	579	2.4627	0.577056	0.506881	4.12231	3.94473	12	12
gvi	377	830	683	2.4789	0.594211	0.518726	4.15974	3.95251	12	12
pma	338	718	578	2.5195	0.577878	0.487342	4.09658	3.92037	12	12
pmm	342	760	614	2.5447	0.590459	0.48967	4.06937	3.92196	10	11
pmt	352	791	626	2.4855	0.581159	0.495484	4.09455	3.98362	12	12
syn	387	823	681	2.5176	0.590339	0.501971	4.1349	3.91225	12	12
tel	350	653	585	2.6718	0.593009	0.488283	4.11994	3.87589	11	12

CC: clustering coefficient; AL: average path length.

**Table 2:** Change of topological properties with randomly reducing size in E. coli metabolic network.

Topological properties	Whole network	5% reduced network	15% reduced network	25% reduced network	35% reduced network	50% reduced network
enzyme CC	0.584591	0.584634	0.593573	0.588487	0.599161	0.598578
compound CC	0.494804	0.498944	0.51094	0.515453	0.523472	0.530856
enzyme AL	4.1142	4.14179	4.20145	4.21134	4.24669	4.31382
compound AL	4.11805	4.14388	4.23782	4.28722	4.34033	4.30933
enzyme diameter	12	12	12	12	12	13
compound diameter	12	12	12	12	12	13
